# Inhibitory effects and underlying mechanisms of *Artemisia capillaris* essential oil on melanogenesis in the B16F10 cell line

**DOI:** 10.3892/mmr.2022.12629

**Published:** 2022-02-04

**Authors:** Min Jae Kim, Elsayed A. Mohamed, Da Som Kim, Mi-Jin Park, Byoung-Jun Ahn, Eui-Bae Jeung, Beum-Soo An

**Affiliations:** 1 Department of Biomaterials Science (BK21 FOUR Program), College of Natural Resources and Life Science/Life and Industry Convergence Research Institute, Pusan National University, Miryang, Gyeongsangnam-do 50463, Republic of Korea; 2 Department of Genetics, Assiut University, Assiut 71526, Egypt; 3 Division of Forest Industrial Materials, Department of Forest Products and Industry, National Institute of Forest Science, Seoul 02455, Republic of Korea; 4 Laboratory of Veterinary Biochemistry and Molecular Biology, College of Veterinary Medicine, Chungbuk National University, Cheongju, Chungbuk 28644, Republic of Korea

**Keywords:** *Artemisia capillaris*, melanogenesis, skin whitening, microphthalmia-associated transcription factor, tyrosinase, tyrosinase related protein 1 and 2

## Abstract

The present study investigated the anti-melanogenic activity of 10 essential oils using the B16F10 cell model. Initially, a wide range of concentrations of these essential oils were screened in order to determine their toxicity levels. The assigned non-toxic concentrations of the tested essential oils were then used to evaluate their effects on melanogenesis. The effects of the essential oils with potent anti-melanogenic activity on cell proliferation, protection against H_2_O_2_-induced cell death and the expression of certain melanogenesis-related genes, including MITF, tyrosinase, tyrosinase related protein (TRP)-1 and TRP-2 were also evaluated. The results revealed that the essential oils extracted from *Citrus unshiu, Juniperus chinensis* L., *Zanthoxylum piperitum* and *Artemisia capillaris* (*A. capillaris*) inhibited melanogenesis. However, among these four extracts, only *A. capillaris* extract enhanced cell proliferation, exhibited anti-H_2_O_2_ activities and decreased the expression level of TRP-1. It was demonstrated that *A. capillaris* extract inhibited melanin synthesis via the downregulation of the TRP-1 translational level. These essential oil extracts, particularly that of *A. capillaris*, may thus be used as natural anti-melanogenic agents for therapeutic purposes and in the cosmetic industry for skin whitening effects with beneficial proliferative properties. However, further studies using *in vivo* models are required to validate these findings and to examine the effects of these extracts on various molecular pathways.

## Introduction

Melanogenesis is a crucial physiological process that occurs in melanocytes by which the melanosomes that synthesize and store melanin pigment are loaded with melanin and are translocated into the epidermal keratinocytes (1). Melanin biosynthesis is a tightly regulated process, with different pathways controlled by several enzymes and regulators. Tyrosinase is the main enzyme, initiating and regulating melanogenesis. However, tyrosinase-related protein (TRP)-1 and −2 are eumelanogenic enzymes contributing to the completion of the process and acting as modifiers for pathway velocity. TRP-1 and TRP-2 stabilize tyrosinase activity and TRP-1 possibly maintains melanosome structural integrity. Moreover, several regulators are involved in melanin biosynthesis, such as microphthalmia-associated transcription factor (MITF), which is considered the main transcriptional regulator of melanogenesis, functioning as the ‘central switchboard’ for the routing of various signals involved in the expression of melanogenesis-related genes (2,3).

Melanin pigment plays a crucial role in the protection of epidermal cell DNA from solar ultraviolet radiation damage, and in determining skin, hair and eye color (4,5). Furthermore, it can modulate skin immune responses and serves as a scavenger of reactive oxygen species (ROS), cellular toxins and miscellaneous chemical compounds, preventing further skin damage (6,7). The excessive reduction in melanin production (hypopigmentation) is associated with abnormal melanocyte development and dysfunction (8,9), which subsequently reduces protection from harmful UV radiations present in sunlight. On the other hand, the aberrant excessive production and the accumulation of melanin (hyperpigmentation) can lead to the development of skin disorders, such as melasma, post-inflammatory hyperpigmentation, solar lentigo, ephelides and café-au-lait macules (10). Moreover, the overproduction of melanin is recognized not only as a pathological concern, but also as a cosmetic issue. In this regard, individuals from a number of countries in Asia, Africa, South America and the Middle East have decided to reduce skin pigmentation to obtain a lighter skin tone, as fair skin is considered synonymous with youth, health, wealth and beauty in different cultures (11,12). However, hyperpigmentation may be congenital as a result of skin issues/systemic disease or it may be caused by environmental factors (13).

The active agents used to suppress melanin production and lighten the skin for therapeutic or cosmetic purposes are either natural or synthetic, and may function at various levels during melanogenesis. However, several of these agents have undesired adverse effects, such as irritation, rashes, inflamed skin, itchiness, toxicity and pain (14–17), and some of these agents exhibit relatively poor skin permeability (17,18). Therefore, there is a need for new safe and effective skin depigmenting agents to overcome these issues. The use of natural products, including essential oils as functional ingredients in cosmetics and depigmenting agents, has received increasing attention due to the growing interest of consumers in ingredients from natural sources. Moreover, several of these products have multiple pharmacological activities, including anti-melanogenic activity (19,20).

The aim of the present study was to identify naturally-sourced agents for skin whitening purposes and for use in the cosmetic industry with beneficial proliferative properties. For this purpose, the anti-melanogenic activity of essential oils extracted from 10 medicinal plants was evaluated using the B16F10 melanoma cell line by measuring the melanin content. The effects of essential oils with potent anti-melanogenic activity on cell proliferation, protection against H_2_O_2_-induced cell death, and the expression of certain melanogenesis-related proteins, namely MITF, tyrosinase, TRP-1 and TRP-2, were also evaluated.

## Materials and methods

### Extraction of essential oils

The hydrodistillation method was used to extract the essential oils from 10 medicinal plants using different plant parts (National Institute of Forest Science; Republic of Korea) (Table I). In brief, 1 kg of the plant part was mixed with 10 liters of distilled water and heated at 102°C using a heating mantle (cat. no. MS-DM608; Misung Scientific Co., Ltd.). The volatile steam was then condensed using a Dean-Stark trap (National Institute of Forest Science; Republic of Korea), and the acquired whole essential oil was dehydrated using anhydrous sodium sulfite and stored at 4°C until use.

### Cells and cell culture

B16F10 mouse melanoma cells (Korean Cell Line Bank) were cultured in Dulbecco's modified Eagle's medium (DMEM; Welgene Inc.) with or without phenol red supplemented with 10% fetal bovine serum (FBS; Sigma-Aldrich; Merck KGaA) and 1% streptomycin/penicillin (Welgene Inc.) under standard culture conditions for 24 h for recovery. The cultured cells were then treated with the assigned concentrations of the tested essential oils for a further 24 or 72 h (Table II) for further analysis. For cell viability assay and western blot analysis, the B16F10 cells were cultured in DMEM in 24-well plates at a density of 55×10^4^ and 3×10^5^ cells/well, respectively, and treated with the essential oils for 24 h. For the 5-bromo-2-deoxyuridine (BrdU) and melanin quantification assays, the B16F10 cells were cultured in DMEM without phenol red in 6-well plates at a density of 1×10^5^ cells/ml and treated with the essential oils for 24 or 72 h, respectively.

### Cell viability assay and determination of half maximal inhibitory concentration (IC_50_) values

MTT assay was used to construct a cell viability curve and to determine the IC_50_ values. The cultured cells were incubated with various concentrations of the essential oils for 24 h. After treatment, the medium containing essential oils was replaced with a solution of 5 mg/ml MTT (Sigma-Aldrich; Merck KGaA) and incubated at 37°C for 2 h. The optical density (OD) was measured at 570 nm using a microplate reader (BioTek Inc.). Cell viability was calculated using the following formula: OD sample/OD control ×100 for each concentration. The cell survival curve and the IC_50_ value for each treatment were calculated from these values using the SigmaPlot software program (V. 10.0; Systat Software, Inc.).

### Measurement of melanin content

For the melanin quantification assay, the cells were pre-incubated with 200 nM α-melanocyte-stimulating hormone (α-MSH; Sigma-Aldrich; Merck KGaA) for 1 h at 37°C before adding the essential oils to promote melanin production. The assigned tested essential oils at non-toxic concentrations (Table II) or anti-melanogenic agent arbutin (250 µM) were added to the culture medium and incubated for a further 72 h at 37°C. α-MSH was used without essential oils as a positive control. DMSO alone was used as a standard control. Following treatment, the extracellular melanin content in 200 µl of culture media was measured using a microplate reader using a microplate reader (Agilent Technologies, Inc.) at 405 nm. To measure the intracellular melanin content, the cells were washed twice with phosphate-buffered saline (PBS) and collected using trypsinization. Centrifugation at 20,000 × g for 15 min at 4°C was performed, and the melanin pellets were dissolved in 1 N NaOH containing 10% DMSO for 1 h at 60°C. The mixed homogenate (100 µl) was placed in a 96-well microplate, and the OD values were measured using a microplate reader (Agilent Technologies, Inc.) at 405 nm. The extracellular and intracellular melanin contents per well were calculated and expressed as a percentage of the control.

### Assessment of inhibitory effects of essential oil extracts on tyrosinase activity

Mushroom tyrosinase activity assay was performed according to the manufacturer's recommendations. In a 96-well plates, 20 µl mushroom tyrosinase (2,000 U/ml, Sigma-Aldrich; Merck KGaA), 30 µl essential oil extracts or arbutin (Sigma-Aldrich; Merck KGaA) as a positive control, 210 µl phosphate buffer (0.1 M; pH 6.8) and 40 µl tyrosine (1.5 mM, Sigma-Aldrich; Merck KGaA) were mixed and incubated at 37°C for 20 min. The OD value was then measured using a microplate reader (Agilent Technologies, Inc.) at 490 nm. The tyrosinase activity in the samples were expressed using the following formula: OD sample/OD control ×100.

### Measurement of cell proliferation

BrdU assay was carried out using a cell proliferation ELISA BrdU kit (Roche Diagnostics) according to the manufacturer's recommendations. In brief, following the treatment period with the assigned concentrations of the essential oils, 100 µl BrdU solution (100 µM) were added to each well in 1 ml medium, and the plates were then incubated at 37°C for 4 h. Subsequently, the cells were fixed using 1 ml FixDenat (Roche Diagnostics) in each well for 30 min and incubated with the kit-supplied anti-BrdU antibody (1:100; Roche Diagnostics) for 90 min at room temperature. After washing, the cells were incubated with 500 µl substrate for 20 min at room temperature, and 125 µl H_2_SO_4_ (1 M) were added. The plates were analyzed at 450 nm using a spectrometer (Agilent Technologies, Inc.).

### Assessment of the protective effects of the essential oils against H_2_O_2_-induced cell death

B16F10 cells were cultured in DMEM containing the assigned concentrations of the tested essential oils (Table II) for 24 h, as described above. H_2_O_2_ (Sigma-Aldrich; Merck KGaA) was then added at a final concentration of 400 µM for 4 h. Cell viability was assessed using MTT assay as aforementioned.

### Western blot analysis

Protein samples from the B16F10 cells treated with the assigned concentrations of the essential oils for 24 h were extracted using Pro-Prep solution (iNtRON Biotechnology Inc.) according to the manufacturer's protocol. The concentration of protein was determined by performing a bicinchoninic acid assay. Subsequently, 5 µg protein were loaded and separated using sodium dodecyl sulfate-polyacrylamide gel electrophoresis on 8–10% gels and transferred onto nitrocellulose membranes (Daeillab Lab Service Co., Ltd.) using the wet transfer system. The membranes were blocked for 2 h with 5% skimmed milk (BD Biosciences) in PBS with 0.05% Tween-20 (PBST) at room temperature. Subsequently, the membranes were incubated with antibodies against MITF (1:300; cat. no. sc-56725; Santa Cruz Biotechnology, Inc.), tyrosinase (1:300; cat. no. sc-20035; Santa Cruz Biotechnology, Inc.), TRP-1 (1:300; cat. no. sc-25543; Santa Cruz Biotechnology, Inc.), TRP-2 (1:300; cat. no. sc-25544; Santa Cruz Biotechnology, Inc.) and β-actin (1:3,000; cat. no. #4967; Cell Signaling Technology, Inc.), which served as an internal control overnight at 4°C, followed by incubation with horseradish peroxidase-conjugated secondary antibodies (1:5,000; cat. nos. ADI-SAB-100 and ADI-SAB-300; Enzo Life Science Inc.) in 5% skimmed milk in PBST for 1 h at room temperature. Luminol reagent (Bio-Rad Laboratories, Inc.) was used to visualize antibody binding. The blots were scanned using Gel Doc 1000, version 1.5 (Bio-Rad Laboratories, Inc.), and band intensities were normalized to β-actin levels.

### Statistical analyses

Data are presented as the mean ± standard deviation (SD). Data were analyzed using one-way analysis of variance (ANOVA) with SPSS 10.10 standard version (IBM Corp.). Means obtained from three independent experiments were evaluated using one-way ANOVA and Tukey's post hoc t-test for multiple comparisons. A value of P<0.05 was considered to indicate a statistically significant difference.

## Results

### Effects of the tested essential oils on cell viability

**Figure 1.**
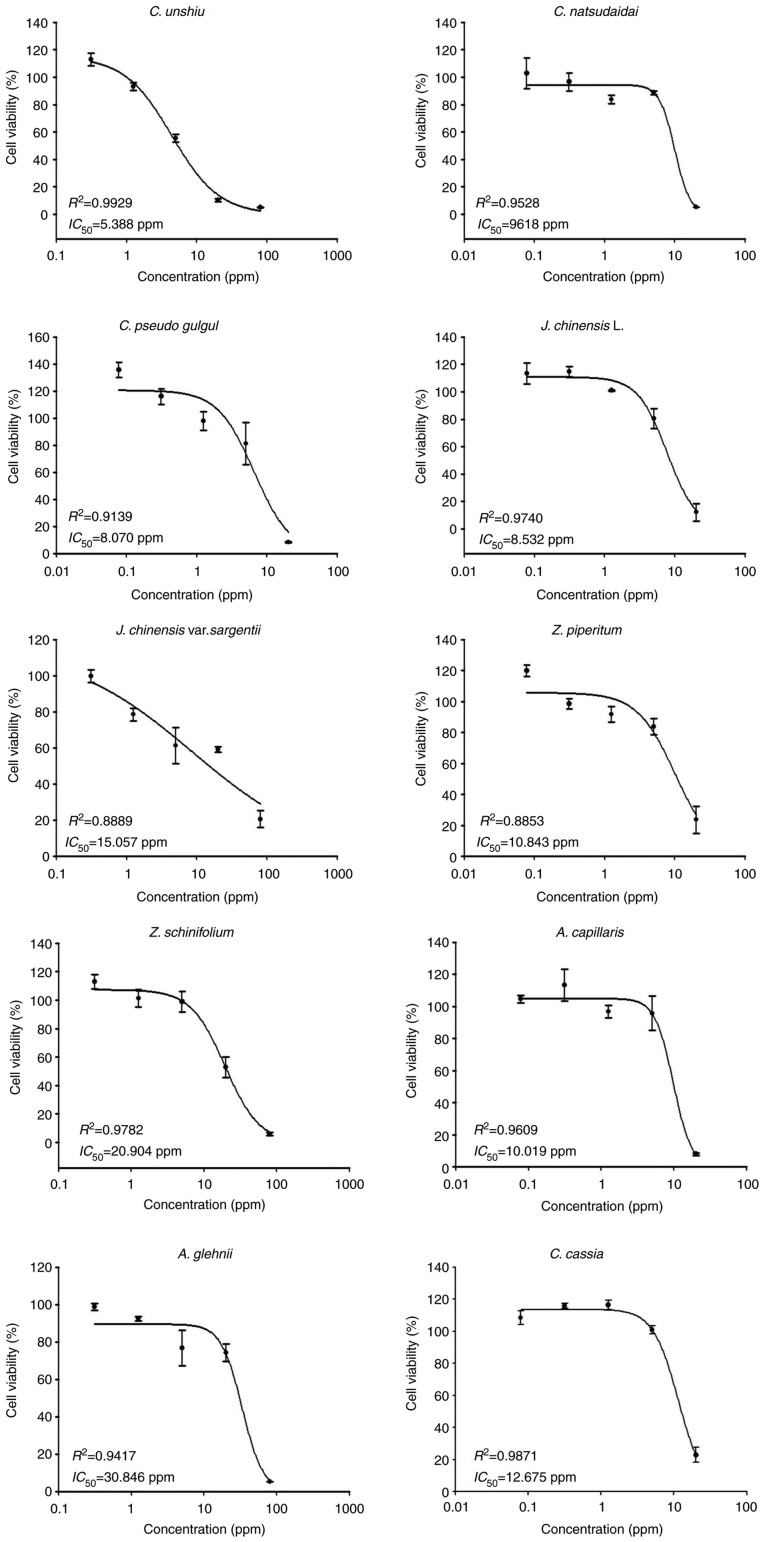
Cell viability curves and IC_50_ values of the tested essential oils. IC_50_, the concentration of the essential oil in the culture media that killed 50% of the B16F10 melanoma cells.

Cell viability assays were conducted using a wide range of concentrations (0.31–80 ppm) for the essential oils extracted from *Citrus unshiu* (*C. unshiu*), *Juniperus chinensis* var. *sargentii* (*J. chinensis* var. *sargentii*), *Zanthoxylum schinifolium* (Siebold & Zucc) (*Z. schinifolium*) and *Aster glehnii* F. Schmidt (*A. glehnii*), and from 0.08 to 20 ppm for the essential oils extracted from *Citrus natsudaidai* Hayata (*C. natsudaidai*), *Citrus pseudo gulgul* (*C. pseudo gulgul*), *Juniperus chinensis (J. chinensis* L.), *Zanthoxylum piperitum* (*Z. piperitum*), *Artemisia capillaris* (*A. capillaris*) and *Cinnamomum cassia* (*C. cassia*) to screen their toxic effects on B16F10 cells. The tested essential oils exhibited variable toxicity levels in the B16F10 cells, as revealed by the cell viability curves and IC_50_ values (Fig. 1). The essential oils extracted from *C. unshiu* exhibited the highest toxicity level (IC_50_, 5.388 ppm), whereas the essential oil extracted from *A. glehnii* exhibited the lowest toxicity level (IC_50_, 30.846 ppm) (Fig. 1).

### Effect of essential oils on melanin content

**Figure 2.**
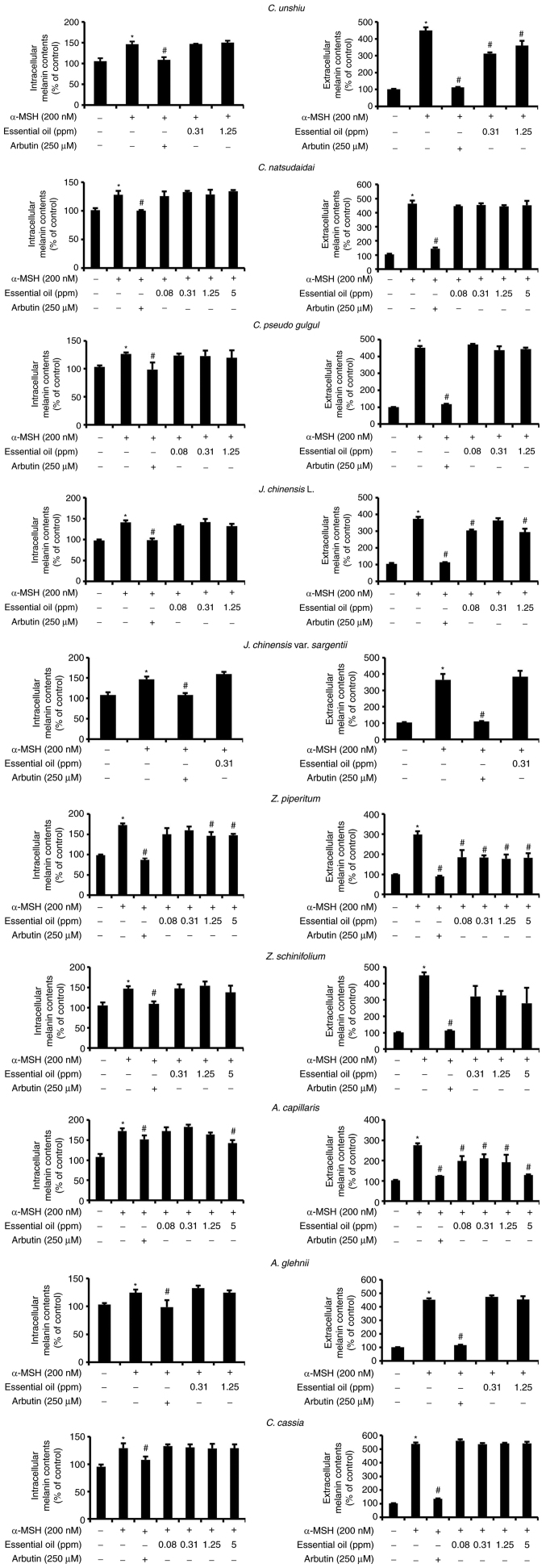
Intracellular and extracellular melanin contents measured following the indicated treatments of the B16F10 melanoma cells. Each value for melanin contents was calculated relative to that of the control group. Values represent the mean ± SD of three independent experiments. *P<0.05, significant difference between the control and α-MSH group; ^#^P<0.05, significant difference compared to the group treated with α-MSH only. α-MSH, α-melanocyte-stimulating hormone.

**Figure 3.**
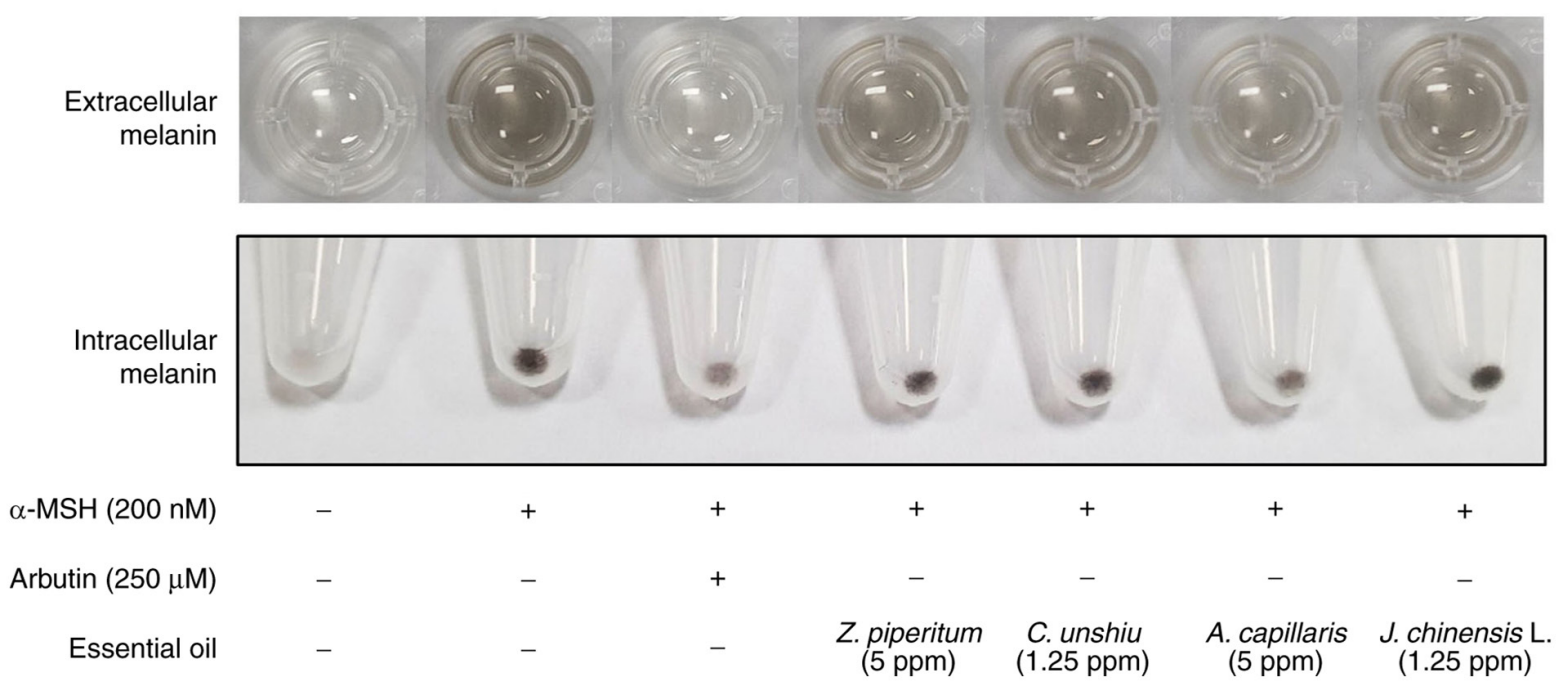
Effects of *C. unshiu, J. chinensis L., Z. piperitum* and *A. capillaris* extracts on α-MSH-induced melanogenesis. B16F10 Cells were incubated with the assigned concentrations of the essential oils extracts or arbutin (250 µM) in the presence of α-MSH (200 nM) for 72 h, collected in a microfuge tube, and photographed. Experiments were performed three times with similar results, and a typical image is presented. α-MSH, α-melanocyte-stimulating hormone.

A set of non-toxic concentrations of the tested essential oils was used to investigate their anti-melanogenic activity in B16F10 cells. The extracellular and intracellular melanin contents were quantified after culturing the cells with the assigned treatments for 72 h (Fig. 2). The extracellular and intracellular melanin contents were significantly higher (P<0.05) in the positive control group (α-MSH) than in the other treatment groups. However, arbutin treatment significantly decreased the extracellular and intracellular melanin content compared to that in the positive control group (P<0.05). Of note, the essential oils extracted from *C. unshiu, J. chinensis* L., *Z. piperitum* and *A. capillaris* significantly decreased the extracellular melanin contents at all concentrations tested compared to the positive control group (P<0.05), excluding the concentration of 0.31 ppm for *J. chinensis* L. The highest concentrations of these essential oils decreased the extracellular melanin content by 20, 38.8, 21 and 53.5% compared with the positive control group, respectively. However, only the elevated concentrations of *Z. piperitum* and *A. capillaris* significantly decreased the intracellular melanin content (P<0.05) compared to that in the positive control group by 14.4 and 17.5%, respectively (Figs. 2 and 3). Of note, none of these essential oils significantly altered the tyrosinase activity (Fig. S1).

### Effect of essential oils on B16F10 cell proliferation

**Figure 4.**
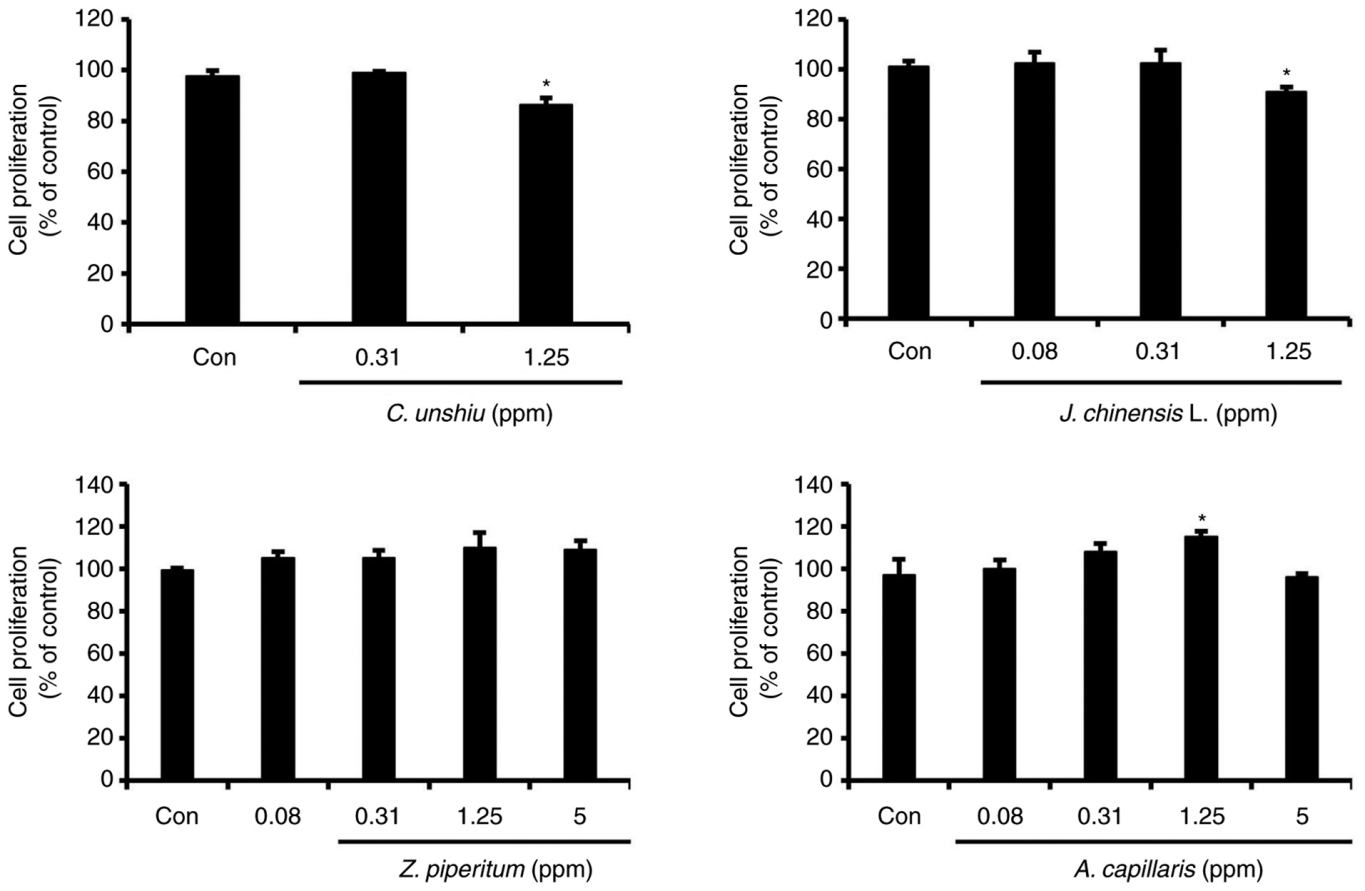
Effects of the essential oils on B16F10 melanoma cell proliferation. *P<0.05, significant difference compared to the control.

Among the 10 essential oils examined in the present study, only four essential oils with anti-melanogenic activity (*C. unshiu, J. chinensis* L., *Z. piperitum*, and *A. capillaris*) were selected to investigate their effects on cell proliferation using BrdU assay. In general, the essential oils extracted from *Z. piperitum* and *A. capillaris* enhanced cell proliferation, although only *A. capillaris* extract at a concentration of 1.25 ppm significantly (P<0.05) increased cell proliferation by 18.7% compared to that in the control group. However, the highest concentrations tested for the *C. unshiu* and *J. chinensis* L. extracts significantly decreased cell proliferation by 11.5 and 10.1%, respectively (P<0.05; Fig. 4).

### Assessment of the protective effects of the essential oils against H_2_O_2_-induced cell death

**Figure 5.**
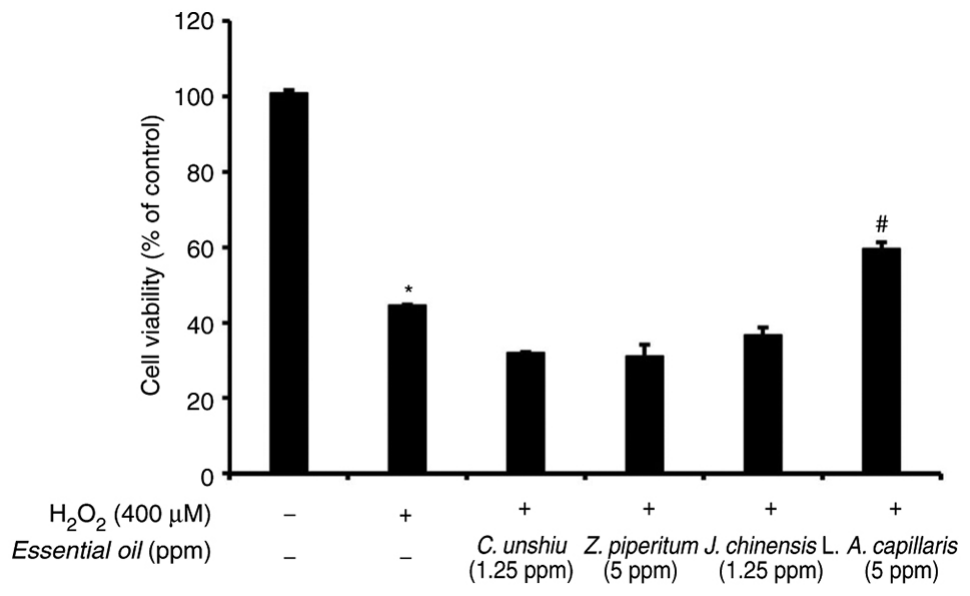
Protective effects of the essential oils against the H_2_O_2_-induced death of B16F10 cells as revealed using MTT assay. *P<0.05, significant difference between the control and the group treated with H_2_O_2_ only; ^#^P<0.05, significant difference compared to the group treated with H_2_O_2_ only.

The protective effects of the essential oils extracted from *C. unshiu, J. chinensis* L., *Z. piperitum*, and *A. capillaris* against H_2_O_2_-induced cell death were assessed using MTT assay. The most effective concentration of the essential oils for an anti-melanogenic effect was used for MTT assay. As shown in Fig. 5, only the essential oil extracted from *A. capillaris* attenuated the effects of H_2_O_2_ on cell death induction and significantly increased cell viability in the presence of H_2_O_2_ in the culture media compared to the other essential oils (P<0.05).

### Effects of the essential oils on the translational levels of melanogenesis-related genes

**Figure 6.**
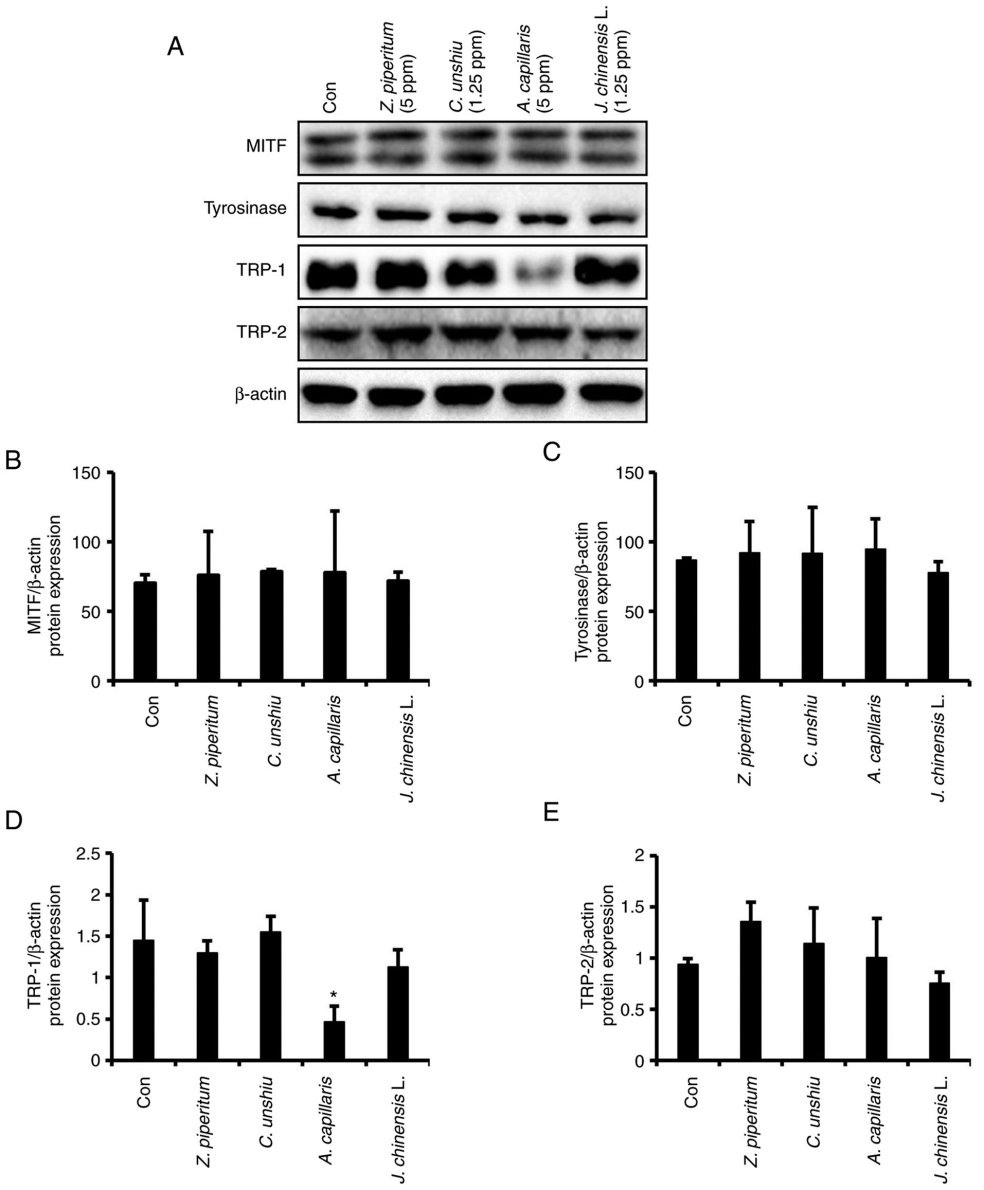
Translational level of melanogenesis-related genes in B16F10 cells treated with different essential oils. (A) Translational levels of melanogenesis-related genes examine using western blot analysis. The expression levels of (B) MITF, (C) tyrosinase, (D) TRP-1, and (E) TRP-2are represented as schematic graphs and normalized to β-actin levels. Data are expressed as the mean ± SD. *P<0.05, significant difference compared to the control. MITF, microphthalmia-associated transcription factor; TRP, tyrosinase related protein.

The effects of the four essential oils extracted from *C. unshiu, J. chinensis* L., *Z. piperitum*, and *A. capillaris* on the MITF, tyrosinase, TRP-1 and TRP-2 translational levels in B16F10 cells were examined using western blot analysis. The most effective concentration of the essential oils for an anti-melanogenic effect was used in western blot analysis. The essential oil extracted from *A. capillaris* at a concentration of 5 ppm significantly decreased the TRP-1 protein level by ~68% compared to that in the control group (P<0.05). However, the other essential oils did not induce any significant changes in the protein levels of tyrosinase, TRP-1 and TRP-2 (Figs. 6 and S2). Among the proteins related to melanogenesis, only MITF exhibited double bands. The upper band has been assigned as a shift of the lower band due to phosphorylation (21). However, none of the essential oils altered the expression levels of MITF (Figs. 6 and S2).

## Discussion

Essential oils extracted from 10 medicinal plants were assessed in the present study to determine their anti-melanogenic activities using B16F10 cell line model. These plants represent various groups of medicinal plants that are widely distributed in a number of Asian countries and have a long history of use in folk medicine to treat various diseases. Moreover, over the past few decades, increased attention has been directed towards the use of functional components from these plants in biomedical applications to treat various diseases, such as cancer (22–25), allergies (26), dermatopathology (27,28) and other diseases (29–32). However, little is known about their effects and functions as natural anti-melanogenic agents.

Melanogenesis is a complex and multistep process that results in melanin formation. Therefore, the measurement of the melanin content is a direct strategy which can be used to assess the melanogenic activity. It has been reported that in an *in vitro* culture system, melanin is synthesized intracellularly and is then transported into the surrounding culture medium (33,34). In the present study, the extracellular and intracellular melanin contents were determined simultaneously to measure the total amount of melanin synthesized under various treatment conditions in B16F10 cells. The anti-melanogenic effects of the essential oils were further examined on the human melanoma cell line, A375SM; however, when treated with α-MSH as a positive control, the cells did not produce a sufficient amount of melanin to evaluate the anti-melanogenic effects (data not shown). Among the essential oil extracts examined, only four essential oils from *C. unshiu, J. chinensis* L., *Z. piperitum* and *A. capillaris* successfully decreased melanogenesis compared to the other extracts, which reflects their potency as anti-melanogenic agents. In agreement with these findings, previous studies have reported the inhibitory effects of some fractions extracted from these four plants on melanin formation using various cell models (31,35–37). Notably, the non-toxic concentrations of all essential oils used in the present study, which inhibited melanin formation, were very low compared to the fraction concentrations used in a previous study, which reached up to 100 µg/ml (36). Although the whole extracts of the essential oils exerted beneficial effects on melanogenesis, active substances were not evaluated in the present study. Therefore, further studies on the active compounds for each essential oil are warranted. However, using the whole essential oils has an advantage over purified components as they have multi-pharmacological activities, and the different components may exert a synergistic or potentiating effects and be important for the bioactivity of the essential oils (38,39). Moreover, unlike previous studies (35–37), the present study used the hydrodistillation method for essential oil extraction, avoiding the hazards of organic solvents and emulsion formation using other methods (40). To v further alidate efficiency of the extracts, their effects on cell proliferation were assessed using BrdU assay, which established the positive effects of two extracts, *Z. piperium* and *A. capillaris*, at specific concentrations on cell proliferation. Additionally, as the skin, more than other tissues, is exposed to numerous external stresses generating several types of ROS, such as H_2_O_2_ that is also produced as a response to a multitude of very complex cellular events causing various deleterious effects and apoptosis in keratinocytes (41,42), the protective effects of *A. capillaris* against H_2_O_2_-induced cell death were also examined. Of note, *A. capillaris* was also able to significantly reduce H_2_O_2_-induced cell death, suggesting that it is an anti-melanogenic agent with proliferative and antioxidant properties. Hong *et al* (43) reported that ethyl acetate fraction from *A. capillaris* exerted significant ROS scavenging and protective effects against oxidative DNA.

The anti-melanogenic effects of the extracted essential oils on the B16F10 cell line were also investigated at the molecular level by assessing the MITF, tyrosinase, TRP-1, and TRP-2 protein expression levels using western blot analysis. Although the four essential oils tested successfully decreased the synthesized melanin content, they did not affect the expression levels of MITF, tyrosinase and TRP-2 proteins, or even tyrosinase activity, and only the essential oil extracted from *A. capillaris* significantly decreased the expression level of TRP-1 compared to that in the control group. Although tyrosinase is the key enzyme in melanogenesis, TRP-1 is considered an eumelanogenic enzymes with a vital role in the completion of melanogenesis. TRP-1 is a protein producing eumelanin in the last stage of the melanogenesis. Eumelanin is the most common type of melanin comprising cross-linked 5,6-dihydroxyindole (DHI) and 5,6-dihydroxyindole-2-carboxylic acid (DHICA). TRP-1 induces the oxidative conversion of DHICA to indole-5,6-quinone-2-carboxylic acid, which is a structural unit of eumelanin (2,3). Therefore, the overexpression of TRP-1 causes skin color to darken (44). Eberle *et al* (45) examined the expression levels of tyrosinase family genes in melanoma and normal melanocyte human cell lines and found that the expression levels of tyrosinase and TRP-2 are regulated differently than TRP-1. Accordingly, in the present study, *A. capillaris* at a concentration of the 5 ppm did not affect the MITF, tyrosinase and TRP-2 expression levels or tyrosinase activity, whereas it significantly decreased TRP-1 expression compared to that in the control group. It appears that *A. capillaris* inhibits melanogenesis via a tyrosinase-independent pathway. Therefore, any materials or components that suppress TRP-1 expression may affect melanogenesis by reducing the oxidation of DHICA to a carboxylated indole-quinone. The findings of the present study suggest that the low expression of TRP-1 can reduce melanin synthesis. The present study initially examined various concentrations of *A. capillaris* (0.08, 0.13, 1.25 and 5 ppm) on cell viability and melanin synthesis. While the concentrations <5 ppm (0.08, 0.13 and 1.25 ppm) did not affect cytotoxicity, these concentrations did not reduce intracellular melanin contents. The concentration of 5 ppm exerted the optimal effect on melanin synthesis, and significantly decreased the extracellular and intracellular melanin contents by 53.5 and 17.5%, respectively compared to the control group. Therefore, this concentration was used to examine the effect of *A. capillaris* against the protein expression levels of TRP-1. Although the *C. unshiu, J. chinensis* L. and *Z. piperitum* extracts decreased the melanin content, none of them altered the translational level of the proteins involved in melanin synthesis. These extracts appear to exert their effects on melanogenesis via mechanisms different from those of *A. capillaris*. As melanogenesis is a tightly regulated process that includes various enzymes and other factors controlling different pathways, materials that exert an inhibitory effect on any of these factors are anticipated to inhibit melanogenesis.

In conclusion, the essential oils extracted from *C. unshiu, J. chinensis* L., *Z. piperitum* and *A. capillaris* using the hydrodistillation method inhibited melanin synthesis. *A. capillaris* extract was the most potent inhibitor of melanin synthesis, with good potential to enhance cell viability and anti-H_2_O_2_ activity. *A. capillaris* extract inhibited melanin synthesis by downregulating the TRP-1 expression level. The present study did not perform animal experiments to reveal the effects of the essential oils. Further animal studies are thus required to address the systemic effects of the essential oils. However, in general, animal experiments to evaluate the functional effects of certain materials on the skin are prohibited for animal protection. Even though the present study did not determine the effects of the extracts in *in vivo* conditions, these four essential oil extracts, particularly *A. capillaris*, may be considered as natural anti-melanogenic agents with beneficial proliferative properties for the treatment of skin pigmentary disorders and for skin whitening in the cosmetic industry. However, future studies using *in vivo* models are required for further validation and to investigate the effects of these extracts at the molecular level through various mechanisms and pathways.

## Supplementary Material

Supporting Data

## Figures and Tables

**Table I.** List of the scientific names, common names, and parts used of the investigated medicinal plants.

No.	Scientific name	Common name	Parts used
1	*Citrus unshiu*	Satsuma orange	Peels
2	*Citrus natsudaidai* Hayata	Natsumikan	Peels
3	*Citrus pseudo gulgul*	Hill lemon	Peels
4	*Juniperus chinensis* L	Chinese juniper	Leaves
5	*Juniperus chinensis* var. *sargentii*	Sargent juniper	Leaves
6	*Zanthoxylum piperitum*	Japanese pepper	Fruits
7	*Zanthoxylum schinifolium*	Peppertree	Fruits
	(Siebold & Zucc)		
8	*Artemisia capillaris*	Yin Chen Hao	Grass clumps
9	*Aster glehnii* F. Schmidt	Ezo-goma-na^a^	Grass clumps
10	*Cinnamomum cassia*	Chinese cinnamon	Leaves

a Japanese common name.

**Table II.** The concentrations of the tested essential oils used in the different assays in the present study.

		Tested concentrations (ppm)			
		
No.	Essential oil source	Cell viability assay	Melanin quantification assays	BrdU assay	Western blot analysis
1	*Citrus unshiu*	0.31, 1.25, 5, 20 and 80	0.31 and 1.25	0.31 and 1.25	1.25^a^
2	*Citrus natsudaidai* Hayata	0.08, 0.31, 1.25, 5 and 20	0.08, 0.31, 1.25 and 5	-	-
3	*Citrus pseudo gulgul*	0.08, 0.31, 1.25, 5 and 20	0.08, 0.31 and 1.25	-	-
4	*Juniperus chinensis* L	0.08, 0.31, 1.25, 5 and 20	0.08, 0.31 and 1.25	0.08, 0.31 and 1.25	1.25^a^
5	*Juniperus chinensis* var. *sargentii*	0.31, 1.25, 5, 20 and 80	0.31	-	-
6	*Zanthoxylum piperitum*	0.08, 0.31, 1.25, 5 and 20	0.08, 0.31, 1.25 and 5	0.08, 0.31, 1.25 and 5	5^a^
7	*Zanthoxylum schinifolium* (Siebold & Zucc)	0.31, 1.25, 5, 20 and 80	0.31, 1.25 and 5	-	-
8	*Artemisia capillaris*	0.08, 0.31, 1.25, 5 and 20	0.08, 0.31, 1.25 and 5	0.08, 0.31, 1.25 and 5	5^a^
9	*Aster glehnii* F. Schmidt	0.31, 1.25, 5, 20 and 80	0.31 and 1.25	-	-
10	*Cinnamomum cassia*	0.08, 0.31, 1.25, 5 and 20	0.08, 0.31, 1.25 and 5	-	-

a These concentrations were also used to assess the protective effects of essential oils against H_2_O_2_-induced cell death.

## Data Availability

The datasets used and/or analyzed during the current study are available from the corresponding author on reasonable request.
