# Supplementary material for: Inhibitory effects and underlying mechanisms of Artemisia capillaris essential oil on melanogenesis in the B16F10 cell line
Source: Mol Med Rep. 2022 Feb 4;25(4):113. doi: 10.3892/mmr.2022.12629 (PMC8845066; doi:10.3892/mmr.2022.12629)

Figure S1. Effects of the essential oils on tyrosinase activity. Data are expressed as the mean ± SD. *P<0.05, significant difference compared to the control.

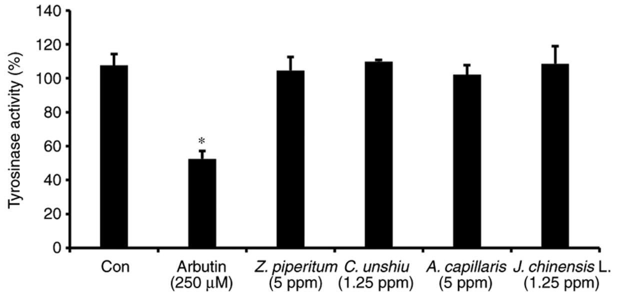

Figure S2. Translational levels of melanogenesis-related genes in B16F10 cells treated with the various essential oils. The second and third replicates of the western blots are shown. MITF, microphthalmia-associated transcription factor; TRP, tyrosinase related protein.

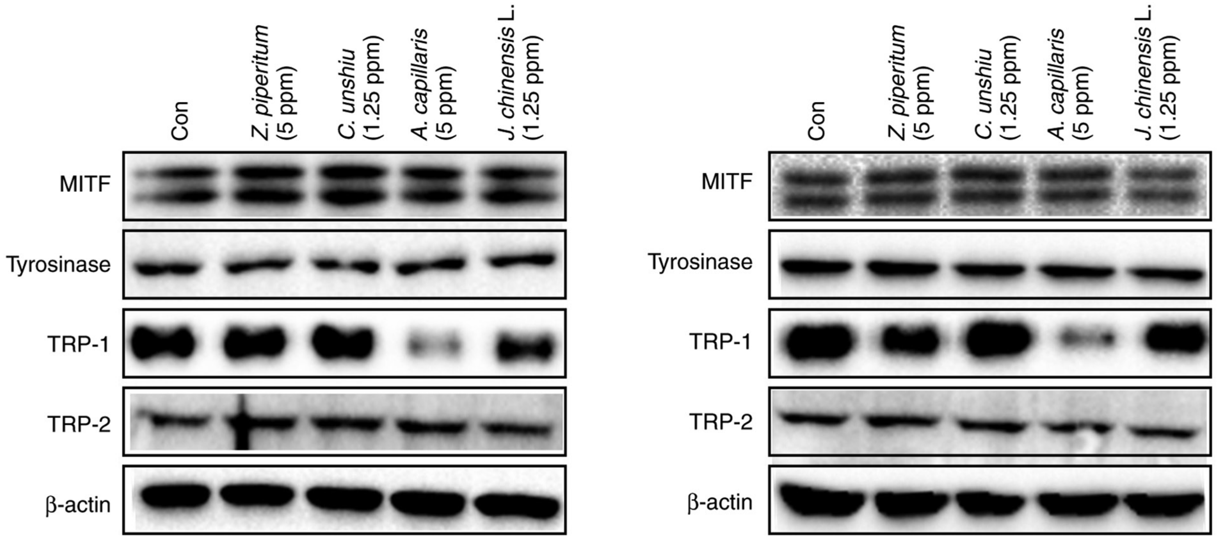

Supplement: Supporting Data [file Supplementary_Data.pdf]
